# Kidney cancer with thyroid metastasis combined with thyroid carcinoma, a case report

**DOI:** 10.1186/s12902-023-01332-3

**Published:** 2023-04-27

**Authors:** Rongli Xie, Dan Tan, Boke Liu, Xujie Han, Xiaoli Jin, Dongjie Shen, Lei Zhou, Jiankang Shen

**Affiliations:** 1 Department of General Surgery, Ruijin Hospital Lu Wan Branch, Shanghai Jiaotong University School of Medicine, Shanghai, China; 2 Department of Urology, Ruijin Hospital, Shanghai Jiao Tong University School of Medicine, Shanghai, China

**Keywords:** Carcinoma, Thyroid cancer, Renal cell, Papillary, Case report

## Abstract

**Background:**

Thyroid cancer is the most common malignant tumor of the endocrine system. There have been some reports on kidney cancer with thyroid metastasis. However, kidney cancer has rarely been detected during thyroid cancer surgery.

**Case presentation:**

We present a rare case of kidney cancer with thyroid metastasis, combined with thyroid carcinoma. A 66-year-old woman was admitted to our hospital in September 2021 due to enlarged left thyroid nodules for two years. The patient was diagnosed with a left thyroid nodule on physical examination in 2012. Extended radical resection of the thyroid cancer was performed. Intraoperatively, two thyroid lesions were identified. Thus, the patient was definitively diagnosed with kidney cancer with thyroid metastasis and papillary thyroid carcinoma. Furthermore, two metastatic nodules due to kidney cancer and one metastatic lymph node lesion due to thyroid cancer were found in the loose connective tissue adjacent to the thyroid.

**Conclusions:**

Kidney cancer with thyroid metastasis and thyroid carcinoma rarely co-occur, and it is difficult to identify the primary tumor. Although clinical examination methods are increasingly updated, the past medical history and physical examination are still very important.

## Background

Thyroid cancer is the most common malignancy of the endocrine system. The incidence of papillary thyroid cancer has increased rapidly [1, 2]. Surgery plays an important role in the treatment of thyroid patients, while clinical follow-up observation is also feasible, especially for pantiens with low-risk papillary thyroid cancer [3, 4]. Despite the advancements in the preoperative diagnostic tools, papillary thyroid cancer can still be misdiagnosed, even with an ultrasound-guided fine-needle aspiration biopsy, the gold standard [2, 5, 6]. Some patients are preoperatively diagnosed with papillary carcinoma. However, rapid freezing of the pathological specimen yielded a postoperative diagnosis of follicular carcinoma, medullary carcinoma, or even metastatic tumors. Some articles have reported kidney cancer metastases to the thyroid, but kidney cancer is rarely an incidental finding in thyroid cancer surgery [7, 8].We present a rare case of kidney cancer with thyroid metastasis combined with thyroid carcinoma. The patient’s clinical characteristics, which aided in the clinical diagnosis and treatment, are summarized in this study.

## Case presentation

A 66-year-old woman was admitted to our hospital in September 2021 for enlarged left thyroid nodules, lasting two years. The patient was diagnosed with a left thyroid nodule on physical examination in 2012. In the following nine years, the patient developed no symptoms, such as hoarseness of the voice, difficulty breathing, and fever.

**Fig. 1:**
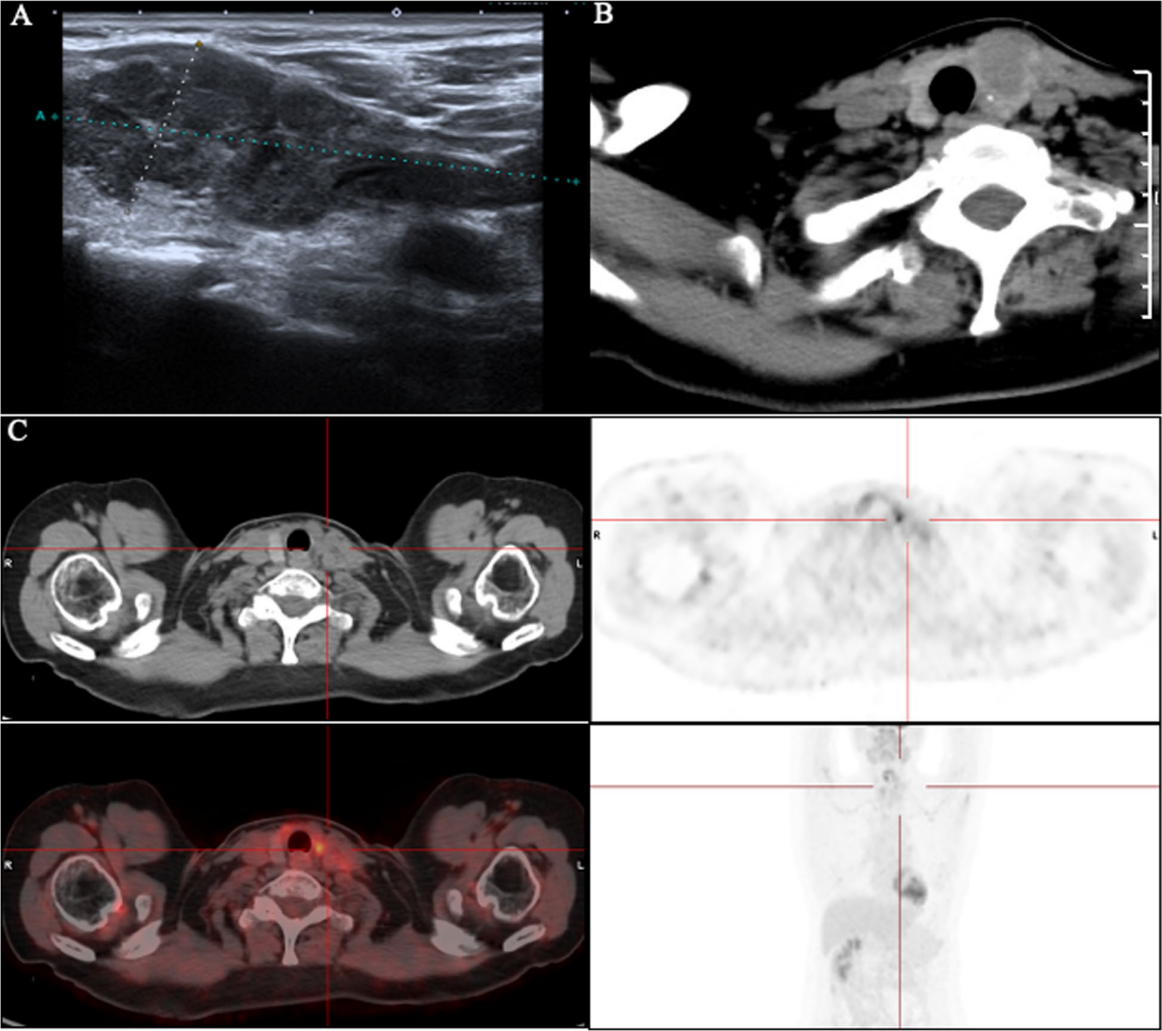
Abnormal imaging findings of patients. **A** The hypoechoic nodule grew horizontally and was composed of fused lesions, measuring 61.6 × 21.4 mm. **B** The preoperative computed tomography (CT) revealed bilateral thyroid nodules with unclear boundaries. **C** Three weeks after the surgery, positron emission tomography-CT revealed increased metabolism in the surgical site of the thyroid

An outpatient ultrasound demonstrated a solid mass in the left thyroid gland (Thyroid Imaging Reporting and Data System 4b). The hypoechoic nodule (shown in Fig. 1A) grew horizontally and was composed of fused lesions, measuring 61.6 × 21.4 mm. It had an irregular shape, poorly defined boundaries, and heterogeneous internal echo. There was no significant internal echo, and no significant changes in the posterior echo. Abundant blood flow signals were appreciated on color doppler flow imaging. Moreover, there were several anechoic, hypoechoic, and mixed echoic nodules (Thyroid Imaging Reporting and Data System 3) in the left and right thyroid glands. One of the upper and middle poles in the left thyroid measured approximately 7.7 × 5.3 mm. One of the lower poles in the right thyroid measured approximately 12.8 × 7.1 mm. Both horizontally growing nodules had an elliptical shape, clear edges, and poor internal echoes. There was no obvious change in the posterior echoes, and the blood supply of color doppler flow imaging was low. Three-dimensional color and power Doppler ultrasound showed no large and twisted blood vessels. There were no apparent abnormalities in the left and right parathyroid regions and cervical lymph nodes.

The preoperative computed tomography (CT) revealed bilateral thyroid nodules with unclear boundaries (shown Fig. 1B). One nodule had a diameter of 2.5 cm, and a small grainy, high-density shadow. Furthermore, the trachea was slightly deviated to the right. No apparent abnormalities were observed in the other neck soft tissues. The laboratory test results (including free triiodothyronine, thyroxine, thyroid-stimulating hormone, parathyroid hormone) were within the normal ranges (Shown Table 1).

**Table 1:** Lab examination of blood

Time	Item	Result	Range
Admission Day	TSH	0.550	0.270-4.200 uIU/ml
	FT3	4.19	3.1-6.8 pmol/L
	FT4	19.41	12.0-22.0 pmol/L
	ATA	0.18	0-9 IU/ML
	TG	0.66	0-4 IU/ML
	Ca	2.60	2.20–2.65 mmol/L
	PTH	51.6	7.00-53.00 Pg/ml

*TSH* Thyroid Stimulating Hormone, *FT3* Free Triiodothyronine, *FT4* Free Thyroxine, *ATA* Anti-thyroid Peroxidase Antibody, *TG* Thyroglobulin, *PTH* Parathyroid Hormone

**Fig. 2:**
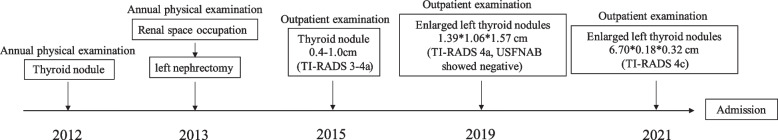
Patient's complete past medical history

The patient underwent a left nephrectomy in 2013, and the postoperative paraffin pathology confirmed clear cell carcinoma (grade II, 5.0 × 4.0 × 3.5 cm). After the kidney surgery, routine outpatient follow-up was performed, without any radiotherapy or chemotherapy. During the clinical follow-up observation, no other metastases were found in other organs. The patient had no history of hypertension, diabetes, and heart disease. The patient's complete past medical history was shown in Fig. 2.

Extended radical resection of the thyroid cancer was conducted (left thyroidectomy, right partial thyroidectomy, and left lateral lymph node dissection). Intraoperatively, two thyroid lesions (measuring 3.5 × 2.5 × 1.5 cm, 0.2 × 0.2 × 0.2 cm, respectively) were found. The former lesion was subsequently confirmed as metastatic kidney cancer by a pathologist. On immunohistochemical examination, the metastatic kidney cancer was CA9( +), Claudin-7(partially positive), CD10 ( +), vimentin ( +), PAX-8( +), PAX-2 (weakly positive), CK19 (partially positive), Galectin-3 ( +), Ki-67 (high expression area 30%), HBME-1 (-), TTF-1 (-), TG (-), CK7 (-), and CD117 (-). The latter lesion was subsequently confirmed as papillary thyroid carcinoma by the pathologist.

Furthermore, two metastatic nodules (0.2–0.4 cm in diameter) secondary to kidney cancer and one metastatic lymph node lesion (0.2–0.4 cm in diameter) secondary to thyroid cancer were found in the loose connective tissue adjacent to the thyroid. Given the patient's tumor stage (Papillary thyroid cancer, T1aN1bM0, ATA intermediate risk), only endocrine therapy (Levothyroxine Sodium Tablets, Euthyrox) was performed. The patient was discharged from the hospital postoperatively. Three weeks after the surgery, positron emission tomography-CT revealed increased metabolism in the surgical site of the thyroid. It was likely caused by metastasis or postoperative inflammatory changes, but it was not determined (shown in Fig. 1C). Based on the tumor characteristics, the patient’s long-term prognosis remains undetermined. However, no thyroid and kidney tumor metastasis has occurred in the patient so far.

## Discussion and conclusions

Renal cell carcinoma (RCC) is a malignant tumor that originates from the inner wall of the proximal tubule. It is the third-most common genitourinary cancer site after prostate and bladder cancer [7]. RCC accounts for approximately 90% of renal tumors, and its pathological types mainly include clear cell carcinoma. Approximately 30% of patients have metastatic disease at the time of RCC diagnosis, and 25% develop metastatic disease following nephrectomy [8]. The most common sites of metastasis are the lungs (75%), followed by the regional lymph nodes (65%), liver (40%), and bone (40%). Thyroid metastasis secondary to RCC rarely occurs [8, 9]. However, RCC is the most common primary tumor in metastatic thyroid cancer [9, 10]. The five-year relative survival for European RCC patients, diagnosed between 2000 and 2007, was 60.6%. This rate was reportedly 72.4% according to the Surveillance, Epidemiology, and End Results program database from 2004 to 2010 [11]. The timing of the onset for thyroid metastasis in RCC is prolonged, and the median time from the diagnosis of RCC to the discovery of thyroid metastasis is estimated to be 8.8 years [12]. Compared to those with other single-site metastases, patients with thyroid metastasis have a better survival after thyroidectomy. Total thyroidectomy was not associated with a better survival than partial thyroidectomy [9]. For the treatment of RCC metastatic to thyroid, the current clinical guidelines do not give clear treatment recommendations. In view of the particularity of this patient, that is, remote metastasis was found by chance eight years after kid surgery, the patient was given Outpatient follow-up after multidisciplinary consultation [13, 14].

In this case, the preoperative examination did not match the final diagnosis. The solid mass in the left thyroid gland (61.6 × 21.4 cm, Thyroid Imaging Reporting and Data System 4b) was considered a primary thyroid cancer. However, the postoperative findings confirmed that it was a a metastasis from RCC. Review the patient's history, the reported thyroid nodes had not been further investigated in 2012, while in 2019 outpatient examination reported an enlarged left thyroid nodule, and it was the same where metastasis of renal cancer was subsequently diagnosed. Another small nodule was identified, and it was diagnosed as a papillary thyroid cancer, instead of a benign tumor. As for the discrepance in size between US and CT, we think it is due to the difference in work principle of machines. The CT scan shows the transverse axis of the mass, so the value is not the maximum diameter. However, on account of the mass effect and the squeezing effect of the sonographer, ultrasound examination may identify the tumor and its surrounding inflammatory tissue as the entire mass, which increase the size of the mass. Hence, both the surgeon and sonographer should take a complete medical history for patients presenting with a thyroid nodule, even though it is a common disease.

Upon reviewing the patient’s previous diagnoses and treatment plans, he was previously diagnosed with a left thyroid nodule in 2012, and he underwent a left nephrectomy (clear cell carcinoma) in 2013. He was admitted to our hospital for enlarged left thyroid nodules two years later. Kidney cancer rarely occurs with thyroid metastasis and thyroid carcinoma. Moreover, it is difficult to identify the primary tumor. This point of medical relevance is worth considering.

## Data Availability

All data generated and analyzed during this study are included in this published article and are available from the corresponding author upon reasonable request.

## Abbreviations

CT: Computed tomography
RCC: Renal cell carcinoma
